# Optimized Tapered Fiber Decorated by Ag Nanoparticles for Raman Measurement with High Sensitivity

**DOI:** 10.3390/s21072300

**Published:** 2021-03-25

**Authors:** Tao Li, Zhinan Yu, Zhengkun Wang, Yong Zhu, Jie Zhang

**Affiliations:** The Key Laboratory of Optoelectronic Technology & System (Ministry of Education), Chongqing University, Chongqing 400044, China; 201808131071@cqu.edu.cn (T.L.); 15682130783@163.com (Z.Y.); 20190801173@cqu.edu.cn (Z.W.); zhuyong@cqu.edu.cn (Y.Z.)

**Keywords:** Raman scattering, tapered fiber, cone angle, AgNPs

## Abstract

A tapered fiber decorated by Ag nanoparticles is prepared as a surface-enhanced Raman scattering (SERS) substrate. There are two key parameters during the preparation process, the fiber cone angle and the density of decorated AgNPs on the fiber tip surface. Their theoretical analysis on the forming mechanism and the optimization process is studied in detail. The tapered fibers with angles from 0.5 to 30° are successfully prepared, with a chemical method in a small tube using a bending interface. AgNPs with different densities are decorated on the surface of the tapered fibers with an electrostatic adsorption method. The optimized tapered fiber SERS probe with an angle of 12° and AgNPs density of 26.67% provides the detection of Rhodamine 6G (R6G) with 10^−10^ mol/L.

## 1. Introduction

Raman spectroscopy is a vibrational spectroscopy technology that can provide molecular fingerprint information for chemical identification. As a spectral analysis method, it has been widely used in various fields, such as chemical analysis [1], material science [2] and biomedical applications [3,4]. However, Raman scattering is extremely weak and it is particularly insufficient when detecting trace substances. Fortunately, various enhanced Raman spectroscopy techniques, including surface-enhanced Raman scattering (SERS) [5,6], waveguide-enhanced Raman scattering [7], resonance Raman spectroscopy [8], tip-enhanced Raman scattering (TERS) [9] and other techniques have greatly improved the sensitivity of Raman scattering. Among them, SERS has a good enhancement effect due to the local surface plasmon resonance (LSPR) characteristics of metal nanostructures. The currently applicable structures include rough metal electrodes [10,11,12,13,14], polymerized thin films [15], metal islands with different morphologies [16,17,18], etc. However, when it comes to flexible detection, in situ monitoring or remote sensing in confined places, the above structures will no longer be suitable. Integrating SERS materials into waveguides can broaden the applications of SERS structures and obtain very high SERS detection repeatability [19,20]. At the same time, the collection efficiency of Raman scattering light is improved by the efficient optical collection effect of waveguide. Therefore, the combination of reverse-symmetry waveguides [21], silicon nitride nanophotonic waveguides [22], optical fibers, etc., and SERS has been greatly developed. Among them, the optical fiber SERS probe combines the advantages of optical fiber dexterity, long-distance transmission and remote online. The currently proposed structures vary from D-type fiber [23] and cylindrical fiber [24,25] to fiber SERS probe reported by us [26].

In this study, we further optimize the tapered fiber angle and the density of AgNPs decorated on the fiber surface, in order to gain higher detection limit. The theoretical analysis on the forming mechanism of different angles, the enhancement of the Raman characteristics, and the improvement of the molecule detection limit are reported.

## 2. Preparation of the Tapered Fiber SERS (Surface-Eenhanced Raman Scattering) Probe

### 2.1. Materials

Multimode fibers (50/125 μm, NA = 0.22) were obtained from Yangtze Optical Fiber and Cable Joint Stock Limited Co., Ltd., Wuhan, China. Rhodamine 6G (C_28_H_31_N_2_O_3_Cl, R6G, 95%), hydrofluoric acid (HF, 49 wt%) and 3-Aminopropyl triethoxysilane (C_9_H_23_NO_3_Si, 99%, APTES) were purchased from Aladdin Biochemical Technology Co., Ltd., Shanghai, China. Sunflower seed oils were purchased from Yonghui Superstores, Fujian, China.

### 2.2. Preparation Process

**Figure 1:**
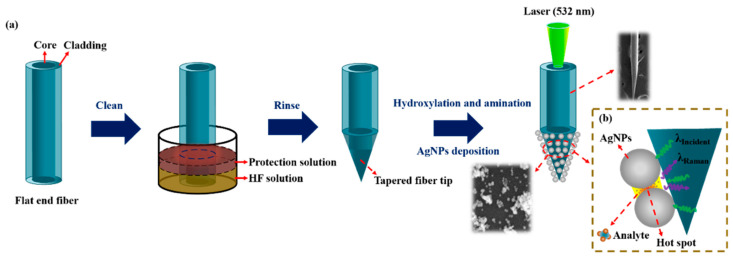
Schematic illustration of (**a**) the fabrication process of the tapered fiber and (**b**) Raman detection.

The preparation process of a tapered optical fiber SERS probe is shown in Figure 1a, which mainly undergoes the following three steps.

Preparation of a tapered fiber: Firstly, remove the coating layer (3 cm from the end of the multimode fiber) by mechanical stripping and clean it with ethanol. Secondly, the exposed fiber was immersed vertically into the mixture of HF acid and sunflower seed oil for fiber corrosion. A sunflower oil layer was used to prevent the non-immersed part from being corroded. After the corrosion was completed, the tapered optical fiber was rinsed with ethanol and vacuum-dried for storage.

Synthesis of AgNPs: AgNPs were prepared as described by Lee and Meisel [27]. Briefly, 34 mg AgNO_3_ in 100 mL deionized water was boiled under continuous stirring. Then, 20 mg sodium citrate was added. The mixture was boiled with stirring for about 35 min. After the solution cooled down to room temperature, in order to remove impurity, Ag colloid was further processed with additional centrifugal (2000 rpm, 45 min) and ultrasonic vibration twice.

Preparation of the SERS probe: AgNPs were deposited on the tip of the tapered fiber by an electrostatic adsorption method. Firstly, the corroded tapered fiber tip was put into a piranha solution (98% concentrated sulfuric acid and 30% hydrogen peroxide, mixed with a volume ratio of 2:1) for 30 min for hydroxylation. Secondly, after washed with deionized water, the fiber tip was put into a preconfigured 10% APTES solution for 90 min for amination, so that the surface of the fiber tip had positive amino functional groups. Thirdly, after washed with deionized water to remove excess chemicals, the fiber tip was immersed into a prepared Ag colloid to adsorb AgNPs through an electrostatic interaction between negatively charged Ag and the positively charged amino functional groups. Silanized fiber surface can accelerate the deposition process of AgNPs and make the fiber attach a large number of AgNPs, which will lead to a stronger SERS enhancement effect and higher detection sensitivity [28].

### 2.3. Characterization

The optical image was taken by an optical microscope (VHX-600, Keyence, Shanghai, China). Scanning electron microscopy (SEM) image was taken by a field emission environmental scanning electron microscope (Quattro S, Thermo Fisher Scientific, Shanghai, China). Raman signals were collected by a Raman spectrometer (LabRAM HR Evolution, HORIBA Scientific, Palaiseau, France), with an excitation wavelength of 532 nm, power of 15 mW (filter is 10%), objective lens of × 10 (numerical aperture (NA) of 0.25 and working distance (WD) of 10.6 mm) and an integration time of 2 s. COMSOL Multiphysics software was used to calculate the distributions of the electrical field.

Rhodamine 6G (R6G) was selected as an analyte molecule to demonstrate the detection capability of our samples. The tapered SERS probe was immersed into R6G solution for 3 min and then taken out of the solution, after natural drying, the probe was taken as the Raman measurement, shown in Figure 1b. The laser was focused on the tapered fiber core through the objective lens and transmitted to the tip of the fiber. Raman signals were transmitted back to the fiber core and collected by the objective lens, recorded by the Raman spectrometer. In order to reduce random error, every Raman data was averaged by multiple detections.

## 3. Experimental Results

### 3.1. Forming Mechanism of the Tapered Angle

**Figure 2:**
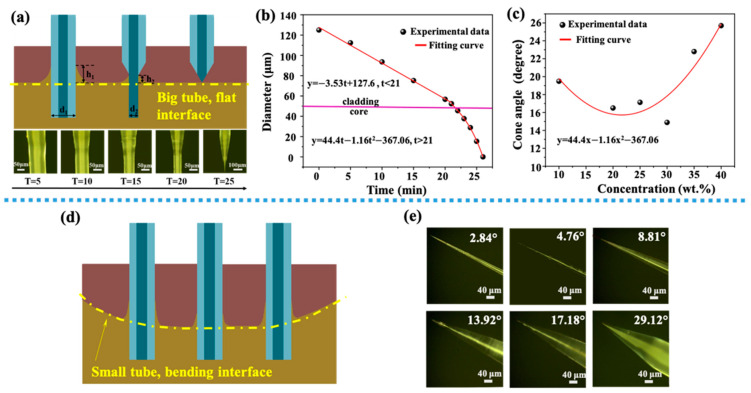
(**a**) Schematic diagram of optical fiber corrosion process (a flat interface), images on the second row show the tip of the fiber at different etching time, with a 5-min interval; (**b**) the relationship between the fiber diameter and the etching time, the red lines are fitting curves for two different stages separated by a horizontal pink line (interface between core and cladding). (**c**) The relationship between HF acid concentration and final cone angle, the red line is a quadratic fitting curve of experimental data. (**d**) Schematic diagram of the meniscus formed by the optical fiber at different positions in a small centrifuge tube (a bending interface). (**e**) Optical images of tapered optical fibers with different angles.

Shown in Figure 2a, when an optical fiber was inserted into a mixed solution of HF acid and protective solution, a meniscus with a height h_1_ was formed at the flat interface of two different solutions. With the progress of corrosion process, the diameter of the optical fiber decreased gradually due to the function of HF. The decrease of the diameter caused the height of the meniscus to decrease to h_2_. When the height of the meniscus reduced to zero, the corrosion process was completed and the final cone angle was formed. The final cone angle was related to some process parameters, including the contact angle, Euler constant, surface tension, liquid density and gravity acceleration constant [29].

In order to study the process of HF acid etching fiber more accurately, as shown in Figure 2a on the second row, optical images show different tapered angles under different corrosion time. The relationship between the fiber diameter and corrosion time is shown in Figure 2b. There were two processes, because the materials of core and cladding were different. The cladding was corroded firstly. When the corrosion time *t* was less than 21 min, HF acid mainly corroded the fiber cladding. When the corrosion time *t* was more than 21 min, HF acid mainly corroded the fiber core, and the diameter decreased sharply. The former process could be fitted by a lineal relationship, *y* = 127.6−3.53*t*. The latter one could be fitted by a quadratic curve. Figure 2c shows the relationship between the concentration of HF acid and the final cone angle. A quadratic function could be used to show their relationship, and a minimal cone angle was gained when HF concentration was 30 wt%. Figure 2d shows the schematic diagram of the meniscus formed when an optical fiber placed at different positions in a small 10 mL centrifuge tube. Since the interface between the protective solution and HF acid solution was a curved surface, this curved surface had an impact on the meniscus generated. When an optical fiber was located in the center of the surface, the height of the meniscus was low, and the corresponding angle of the optical fiber formed by corrosion was a little large. When an optical fiber was located at both ends of the curved surface, the height of the meniscus was high and a little small angle could be formed. Figure 2e shows the optical figures of tapered fiber obtained by etching in a 10 mL centrifuge tube. By this method, a tapered fiber probe with angle distributions of 0.5–30° could be obtained.

**Figure 3:**
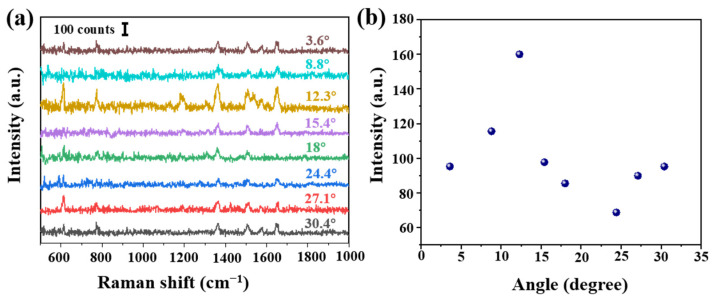
(**a**) Intensity of Raman spectra at different angles (R6G concentration of 10^−6^ mol/L) and (**b**) Raman intensity at 1364 cm^−1^ at different angles. Note that each Raman spectrum is a result of averaged spectra data, in order to delete random error. The standard deviation is less than 10%.

In order to verify the influence of the cone angle on the Raman signal, tapered fibers with different angles were prepared by hydroxylation for 30 min, amination for 90 min and deposition of AgNPs for 30 min. These SERS probes were immersed in 10^−6^ mol/L R6G solution for 3 min, and Raman were carried out after natural drying, shown in Figure 3a. The obvious Raman peaks 611, 774, 1364 and 1650 cm^−1^ were corresponding to the C-C-C ring in-plane bend, C-H out of plane bend, the combination of the four stretching modes and the aromatic C-C stretching vibrations, respectively. Raman intensity at 1364 cm^−1^ at different angles is shown in Figure 3b. We analyzed as follows: (a) the Raman signal increased with the increase of cone angle. The maximal value was obtained at 12.3°. However, Raman signal shows a downward trend, then a slow upward trend, reaching a minimum at 24.4°. (b) On the one hand, we thought different cone angles led to different SERS active regions and numbers of total internal reflections. Calculated cone side surface areas were different at different cone angles, and it decreased with the increase of cone angle. A tapered fiber with a smaller cone angle will provide more active regions and internal reflections, thus allowing more excitation light to interact with SERS materials and target molecules. (c) On the other hand, a smaller angle fiber was easier to damage and more difficult to be saved. Additionally it could be more difficult to deposit AgNPs on a smaller tip. In Section 3.3, we will further carry on a detailed numerical analysis.

### 3.2. Coverage Density of AgNPs

**Figure 4:**
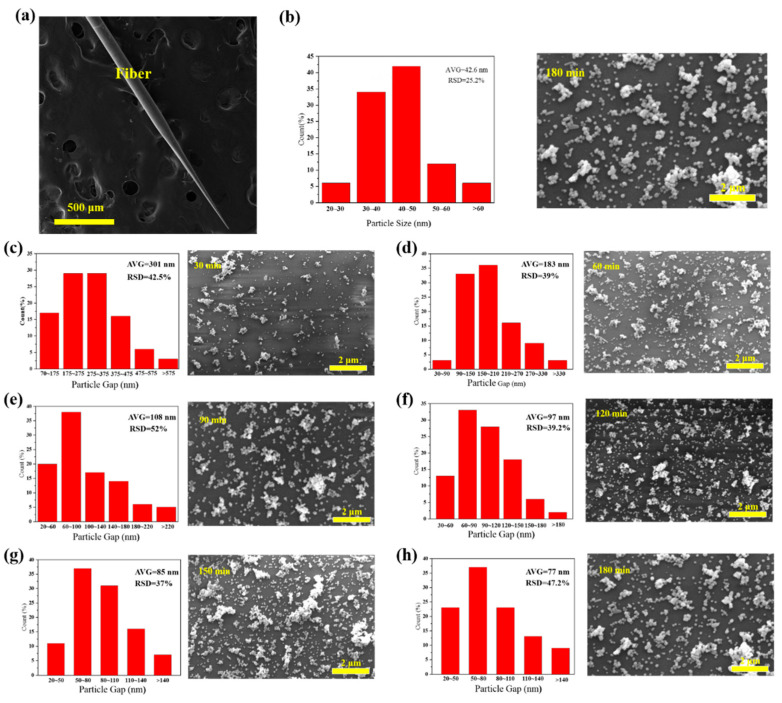
(**a**) SEM (Scanning Electron Microscope) image of a fiber surface-enhanced Raman scattering (SERS) probe (cone angle of 4°); (**b**) size distribution for AgNPs decorated tapered fiber samples and (**c**–**h**) SEM images of different AgNPs deposition time at surface of the tapered fiber tip and corresponding spacing distribution and for 30 min, 60 min, 90 min, 120 min, 150 min and 180 min deposition time, respectively.

**Figure 5:**
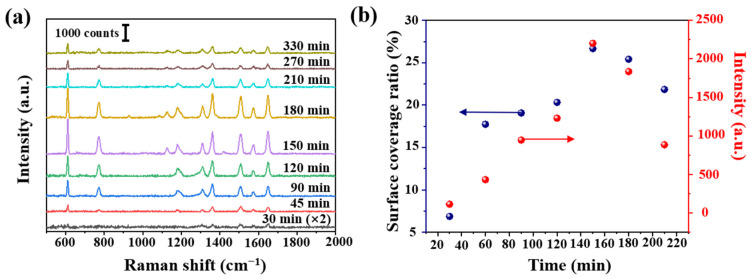
(**a**) R6G Raman intensity (10^−7^ mol/L) at different deposition time and (**b**) calculated Ag surface coverage ratio at different deposition time, and the corresponding Raman intensity at 611 cm^−1^ from (**a**).

We further studied the effect of AgNPs density, using tapered fibers (angle of 4°, hydroxylated for 30 min and aminated for 90 min). The SEM image of a fiber SERS probe is shown in Figure 4a. AgNPs size distribution histogram, interparticle spacing distribution histogram and SEM images of tapered fiber surface with different deposition time are shown in Figure 4b–h. The corresponding R6G Raman signals are shown in Figure 5a. The coverage density of AgNPs was calculated based on SEM images. Raman intensity at 611 cm^−1^ and coverage density induced by different deposition time is presented in Figure 5b. With the increase of deposition time, the surface coverage density of AgNPs began to increase, and the maximal value was 26.67% at a deposition time of 150 min. With the deposition time continues increasing, the surface coverage density began to decrease, which could be due to a multilayer aggregation effect of AgNPs [30]. The corresponding Raman signal intensity had a similar trend, mainly due to a larger coverage area of deposited AgNPs and a smaller gap between nanoparticles, leading to a stronger localized electric field enhancement. We will further carry on numerical analysis in Section 3.3.

### 3.3. Numerical Analysis

**Figure 6:**
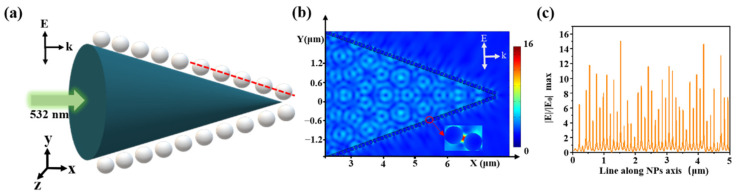
(**a**) Schematic diagram of the simulation model, an incident light of 532 nm, Ag diameter of 100 nm, gap of 10 nm; (**b**) spatial distribution of the electric field and (**c**) the corresponding electric-field values along a red line in (**a**).

Optical simulations were performed using a finite-element method (FEM) with COMSOL Multiphysics. Since the size and gap of AgNPs were nanoscale, and the size of fiber core was micron, we had an extremely large number of mesh elements due to the nanoparticles and nanometer scale gaps between them, it was very difficult to simulate with a 3D model [31]. So, we modeled our SERS probe with a 2D model in Figure 6a. Based on SEM images, we set AgNPs radius of 50 nm, a gap of 10 nm, a cone angle of 40°, and a fiber tip length of 10 μm. The laser wavelength was 532 nm, the vector *k* was along the x-axis, and *E* was along the y-axis with an amplitude of *E*_0_ = 1 *V/m*. The left boundary was defined as a scattering boundary condition. The other boundaries were defined as perfectly matched layers (PMLs) with a 300 nm thickness. The electric field distribution is shown in Figure 6b. The transmit wave excited the LSPR between AgNPs, which generated hot spots in adjacent AgNPs. Taking a cross-section line along the center of the nanoparticle (the red line in Figure 6a, the electric field value distribution is shown in Figure 6c, where the highest electric field of |E|/|E_0_| was 16).

**Figure 7:**
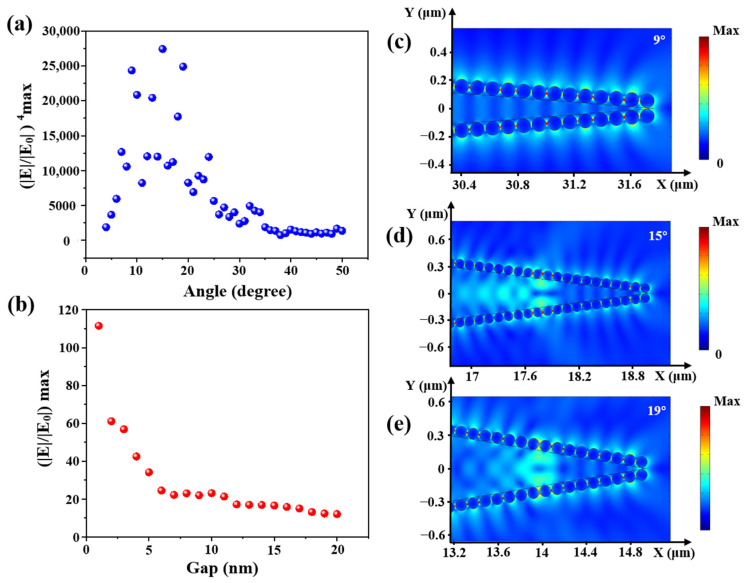
(**a**) The relationship between (|E|/|E_0_|^4^) max and the cone angle; (**b**) the relationship between |E|/|E_0_|max and the gap between two AgNPs and the electric field distribution with cone angles of (**c**) 9°, (**d**) 15° and (**e**) 19°.

Furthermore, the electronic field distributions of cone angles from 4 to 50° with a step of 1° were calculated. The relationship between calculated enhancement factor (EF, (|E|/|E_0_|)^4^ max) and the cone angle is shown in Figure 7a. The electronic field distributions at some obvious angles of 9°, 15° and 19° are shown in Figure 7c–e. EF first increased and then decreased with the increase of fiber cone angle, and there was a large value between 9 and 19°. Compared with Figure 3b the experimental results show that the maximal Raman intensity was obtained at 12.3°. In contrast with the simulation, the practical experiment was affected by more factors, such as the laser focus deviation, the flatness of the fiber input end, etc., which made the experimental results different from the simulation.

In addition, calculated electronic field distribution of different gaps of AgNPs in the range of 1–20 nm with a step of 1 nm is shown in Figure 7b. The smaller the gap between AgNPs, the higher the electric field enhancement. The electric field intensity between two AgNPs was mainly derived from the coupling effect. When the nanogap decreased, the coupling effect would gradually increase. Hence, the intensity of the “hot spot” would increase. Compared with Figure 5b, a higher surface coverage of AgNPs led to a higher Raman intensity.

### 3.4. Time Mapping Experiments

**Figure 8:**
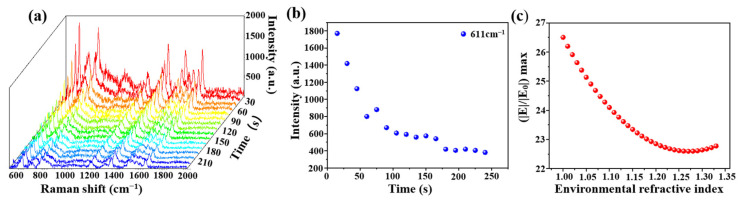
(**a**) Raman time mapping of R6G (10^−6^ mol/L, at a 15 s interval); (**b**) the relationship between Raman intensity at 611 cm^−1^ and testing time and (**c**) the relationship between (|E|/|E_0_|) max and environmental refractive index with the COMSOL simulation.

An optical fiber SERS probe (cone angle of 17°, deposition time of 30 min) was immersed in the R6G solution (10^−6^ mol/L) for 3 min, and then the R6G solution was removed. The Raman signal of R6G was immediately recorded by a Raman spectrometer, shown in Figure 8a. Raman intensity at 611 cm^−1^ at a different time was shown in Figure 8b, there was a decrease along with time and the signal tended to be stable after 175 s. We speculated that this could be a change of an effective refractive index of the molecular solution from a wet state to a dry state. In order to verify this speculation, we calculated the electronic field distributions with different environmental effective refractive indexes from 1.33 (water) to 1 (air) with a step of 0.01. The variation of (|E|/|E_0_|) max with the environmental effective refractive index is shown in Figure 8c. The simulation result was highly similar to that of the experimental result.

### 3.5. Performance of the Optimized Fiber SERS (Surface-Enhanced Raman Scattering) Probe

**Figure 9:**
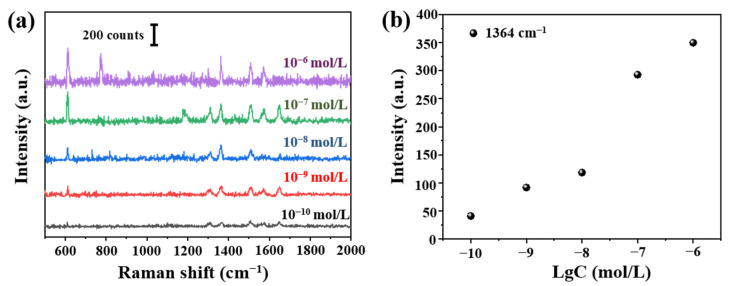
(**a**) Raman spectra from 10^−6^ to 10^−10^ mol/L R6G and (**b**) the relationship between Raman intensity at 1364 cm^−1^ and Lg (R6G concentration).

According to aforementioned optimized tapered fiber angle (12.4°) and deposition time (150 min), we prepared the fiber SERS probes. Raman signals of R6G at different concentrations (10^−6^–10^−10^ mol/L) were shown in Figure 9a. The detection limit was 10^−10^ mol/L. We can see clearly the relationship between Raman signals at 1364 cm^−1^ and concentration in Figure 9b.

The analytical enhancement factor (AEF) was calculated by [32]

(1) $$\mathrm{AEF}=\frac{I_{SERS}/c_{SERS}}{I_{RS}/c_{RS}}$$

where *I_SERS_* and *I_RS_* is the Raman intensity of R6G on the fiber SERS probe and SiO_2_/Si substrate, respectively. *c_SERS_* and *c_RS_* was the immersed concentration of R6G of the fiber SERS probe and SiO_2_/Si substrate, respectively. The calculation result of AEF at 611 cm^−1^ was 5.5 × 10^6^.

Another method to evaluate the enhancement factor (EF) at 611 cm^−1^ of the fiber SERS probe was to calculate the ratio of Raman scattering cross section under the SERS condition and non-SERS condition [33], which was based on the signal of uncoated AgNPs fiber (confirmed as a fluorescent signal in practical measurement), the EF calculated by this method was 3.8 × 10^10^.

## 4. Conclusions

In this paper, a tapered fiber SERS probe was successfully prepared, orderly by forming a tapered fiber with HF acid corroding and AgNPs deposition with an electrostatic adsorption method. The cone angle can be tunable in the range of 0.5–30°. An optimized fiber SERS probe with a cone angle of 12° and AgNPs adsorption density of 26.67% could realize Raman detection of 10^−10^ mol/L R6G. Additionally, the corresponding AEF at 611 cm^−1^ was 5.5 × 10^6^. The EF evaluated by calculating the ratio of Raman scattering cross section under SERS condition and non-SERS condition was 3.8 × 10^10^ at 611 cm^−1^. In addition, compared with the contemporary fiber probe [24,26,34], we increased the detection limit of R6G by two orders of magnitude. The method described in this paper has the potential to be used for remote and online Raman detection of trace molecules. Subsequently, the quantitative detection of tapered fiber SERS probes will be further studied.

## Data Availability

Not applicable.
